# PL-TOON: A Low-Cost Experimental Platform for Teaching and Research on Decentralized Cooperative Control †

**DOI:** 10.3390/s21062072

**Published:** 2021-03-16

**Authors:** Andrés A. Peters, Francisco J. Vargas, Cristóbal Garrido, Cristóbal Andrade, Felipe Villenas

**Affiliations:** 1 Faculty of Engineering and Sciences, Universidad Adolfo Ibáñez, Santiago 7941169, Chile; 2 Departamento de Electrónica, Universidad Técnica Federico Santa María, Valparaíso 2390123, Chile; francisco.vargasp@usm.cl (F.J.V.); cristobal.garridov@sansano.usm.cl (C.G.); cristobal.andradea@sansano.usm.cl (C.A.); felipe.villenas@sansano.usm.cl (F.V.)

**Keywords:** control engineering education, STEM, multi-agent systems laboratory, string stability, vehicle platooning

## Abstract

In this paper, we present the development of a low-cost multi-agent system experimental platform for teaching, and research purposes. The platform consists of train-like autonomous agents equipped with local speed estimation, distance sensing to their nearest predecessor, and wireless communications with other agents and a central coordinator. The individual agents can be used for simple PID experiments in a classroom or laboratory setting, while a collection of agents are capable of performing decentralized platooning with cooperative adaptive cruise control in a variety of settings, the latter being the main goal of the platform. The agents are built from low cost components and programmed with open source software, enabling teaching experiences and experimental work with a larger number of agents that would otherwise be possible with other existing solutions. Additionally, we illustrate with experimental results some of the teaching activities that the platform is capable of performing.

## 1. Introduction

In recent years, advances on control systems and wireless communications technologies have facilitated the implementation of complex multi-agent systems (MAS) applications. Industrial processes, transportation systems, and energy systems, to name a few examples, are among the main areas of impact that can be potentially optimized through the design of control algorithms for systems comprised of several dynamical agents, aiming to work in a coordinated fashion [1,2,3,4,5,6,7]. Given the nature of these type of systems, whose complexity increases with the number of members and interactions, the study of their coordinated behavior is a challenging task, as is the design of appropriate control systems and their implementation. This motivates the development of experimental setups suitable to study such systems and their most relevant aspects, where scalability issues rank as one of the most important.

In this context, scalability refers to the behavior of the MAS when the number of agents increases. A given control system that exhibits an acceptable behavior for a MAS composed of a few agents could cause poor performance for a MAS with a larger number of agents. Scalability is a key aspect in applications such as automated highway systems (AHS), in which a platoon of vehicles are automated to navigate in a highway. In such a scenario, platooning schemes able to achieve a tight formation with a constant speed reference, may suffer from the amplification of disturbances or local errors, as they propagate along the chain of vehicles, inducing performance and safety issues that are intensified as the number of vehicles of the platoon increases [8,9,10]. Such undesirable phenomena is known as string instability.

In this work we are interested in developing an experimental platform for MAS problems where the agents are vehicles that can move in one dimension. Preliminary results in this direction where previously repoted in [11]. In particular we are interested in a platoon of vehicles that autonomously navigates a track in one direction, maintaining a predetermined inter-vehicle distance while traveling at a desired speed. These types of configurations are suitable for modeling AHS where the vehicles will rarely overtake other members of the platoon, unless they are merging or departing from the formation [12,13,14]. The theoretical aspect of such problem has been widely studied, including configurations where string stability is guaranteed. As the study and design of control schemes is of high theoretical complexity, their practical implementation aspects are no less so, and must be considered. Indeed, to achieve the platooning goal, the vehicles must be appropriately equipped with distance sensors, a computing unit that will determine the acceleration or braking actions and a power stage to manipulate the movement. To achieve a string stable configuration with linear control, the agents must posses, at least, either a measurement of their speed or be able to receive the position and/or speed of the platoon leader [15,16,17]. The latter implies the setting up of a communication network to send and receive information among vehicles. Naturally, the implementation costs of real platoons increase as the number of vehicles in the platoon increase.

The implementation aspects of platooning are highly limited due to space and costs restrictions and are normally carried out for a very constrained number of agents. This has motivated the development of lab-scale experimental setups to study the coordinated operation of autonomous agents in higher education institutions and related companies [18]. These setups may vary in their technical capabilities, and also their costs and applicability. For instance, in [19,20], an experimental platform for autonomous multi-agent systems are presented but with no communication capabilities between agents. In [21,22], the agents are not fully autonomous since they are coordinated and controlled by a central processing unit. In [23,24,25] the experimental setups have a high per-agent cost. We find relevant to mention that none of the above works are dedicated to study platooning problems.

In the context of control system laboratories, much effort has been made in recent years to implement robotic kits as tools in aiding with the learning process of secondary and higher educations students (See for instance [26] for a review of modular robotic kits for teachers). A Project-based learning toolkit and Matlab-based applications for teaching, both using quadrotors, were recently explored in [27,28] respectively. The work [29] reports Arduino-based projects for improving automatic control learning. More recently, works such as [30,31] report learning tools that demonstrate fuzzy techniques in a control laboratory project setting.

A large percentage of the works mentioned above are based on low-cost micro-controller units (MCUs), with Arduino boards being the most common; they have become the standard environment in embedded systems development. This is due to their accessibility and the large and active community that has generated a healthy ecosystem for makers and teachers. Within the context of simple autonomous agents, [32] reports an ultra low-cost line follower agent, based on an Arduino board. Basic Arduino boards such as the UNO are easy to use but they usually require extra components to include wireless capabilities or other features. Another widely adopted framework for control laboratories is the one provided by the LEGO^®^, MINDSTORMS^®^, kits. In particular, the EV3 ($350 USD for 2 students) [33] is a commercially available product for building agents that could perform platooning tasks (as they include sensing devices and are able to be programmed). They are capable of interfacing with Matlab/Simulink with native support, with a LEGO^®^, library ready to use. However, this fact does not simplify the implementation of control algorithms. Moreover, their price is not competitive for building a large scale platoon or multi-agent system lab, as it can be attested by the large quantity of similar open platforms developed by researchers and mentioned above. For our platform, we opted for the use of an ESP32 [34] -based MCU that adopts the ubiquitous Arduino UNO footprint, making it familiar for students and compatible with several breakout boards (or Hats) and existing libraries for Arduino boards and similar MCUs. However, when compared to the Arduino UNO, the ESP32 boards at a similar price range offer increased computational power in the shape of a dual core CPU with higher clocks, increased flash memory, and wireless communication capabilities that are fundamental for cooperative platooning schemes.

The contribution of this work is to present a scaled down experimental platform to study platooning. The platform is a train-like setup, where agents are restricted to moving along a rail, and such that each agent is able to measure their own speed, their distance to their immediate predecessor and capable of communicating relevant data to each other, enabling cooperative control schemes. The restricted movement allows focusing on the main issues in platooning problems, while the communication and sensing capabilities allow implementing different control algorithms and interconnection topologies with a decentralized processing [5,35,36]. We also include capabilities such that a third-party device can connect to the platoon agents and perform real-time monitoring of the signals of interest in each agent. The proposed platform is designed with a low budget of less than 30 (usd) per agent, but presenting a high degree of flexibility in its capabilities. This is possible due to recent advances in MCUs, low cost sensors and ad-hoc communication protocols for IoT applications, allowing us to develop low cost embedded systems for non-trivial automated tasks. With the designed platform, we are able to illustrate standard control techniques, such as proportional–integral–derivative (PID) control, in a teaching environment, but also validate theoretical results on the complex aspects of string stability and large platoons problems (such as those in [15,37,38]). A repository with the used MCU codes, scripts for supervision and remote configuration, 3D printed models, figures and videos of the platform in action can be accessed at https://github.com/pl-toon/pl-toon-codes (accessed on 10 March 2021).

The rest of the paper is organized as follows: Section 2 includes the description of the platooning problem that motivates the platform, presenting some theoretical and practical aspects. Materials and methods are in Section 3, which presents the design of the agents that comprise the experimental setup, including technical aspects of their components and capabilities. We also describe here the modules and the functions that the agents are capable of performing and their basic configurations. Section 4 is devoted to the setting up of the platform. In Section 5 we provide comparisons with the most similar platforms to the one presented here. Experimental results are in Section 6, where we propose educational activities that can be performed with the use of the platform. In Section 7 we discuss the scope of the experimental platform and propose research activities to perform. We conclude with some final remarks in Section 8.

## 2. Motivational Multi-Agent Problem

As mentioned before, the experimental setup that we present is motivated by the study of the scalability properties of autonomous mobile agents. We aim to provide an open and affordable platform for science, technology, engineering and mathematics (STEM) subjects education, which is functional and flexible enough to serve as an alternative to professionally built ones for platooning research. Specifically, we are interested in the case where each agent is capable of autonomously navigate in a one dimensional setting, forming a platoon of agents that moves in a coordinated way. Platooning has relevance in cooperative adaptive cruise control problems in AHS.

### 2.1. Platooning Setup

**Figure 1:**
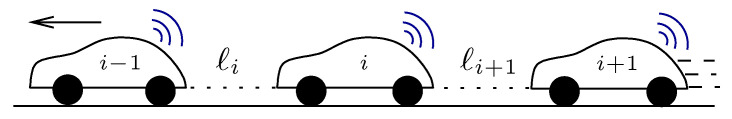
Platoon of vehicles with wireless capabilities.

Using a framework similar to the ones presented in [9,10], we are interested in the formation control problem for a collection of *N* vehicles that travel in a one dimensional track with no overtaking. The position of the *i*-th agent is denoted by $x_{i}\left(t\right)$, while the inter-vehicle distance is given by $\ell_{i}\left(t\right)=x_{i-1}\left(t\right)-x_{i}\left(t\right)$ (see Figure 1). To ease the platooning analysis, researchers commonly assume that the vehicles are identical (homogeneous platoon), with no volume, or length. Also each vehicle dynamic is modeled by a Linear Time Invariant (LTI) system, which is controlled by a local LTI controller. This allows us to use the well-known analytical tools of linear control systems. Consequently, we can use in frequency domain analysis (omitting the Laplace transform arguments (s) for ease of notation) and write

(1) $$\begin{array}{c} X_{i}=H\left(U_{i}+D_{i}\right)\;\mathrm{for}1\leq i\leq N, \end{array}$$

where $X_{i}$ is the Laplace transform of $x_{i}\left(t\right)$, $U_{i}$ is the Laplace transform of the controller output at the *i*-th local controller, $u_{i}\left(t\right)$, $D_{i}$ is a disturbance at the *i*-th agent and *H* denotes the frequency domain dynamics of the LTI model for the vehicles.

The main goal is that each vehicle navigates at the same constant speed, while maintaining a desired inter-vehicle distance respect to its predecessor, and rejecting any possible disturbance. This implicitly requires that the platoon leader implements a type of adaptive cruise control strategy, and that the remaining vehicles reach and maintain the cruise speed within a safe distance.

To achieve such goal, each vehicle uses information of themselves and any other vehicle if available. We assume that each vehicle has access to its instantaneous velocity ${\dot{x}}_{i}\left(t\right)$, its position $x_{i}\left(t\right)$, and the distance to its immediate predecessor $\ell_{i}\left(t\right)$, in addition to other local information as control signals, fuel status, etc. However, when assuming wireless communication capabilities in the platoon, such local information can be transmitted to other vehicles located in any arbitrary position in the chain of vehicles. The shared information among vehicles yields a communication network with a specific topology. The communication topology affects, in general, the performance of the entire platoon. Also, depending on the platoon length, the communication network could be complex and hard to implement, so it is desirable to use a simple network topology but ensuring a reasonable performance.

The platooning problem can be divided in two parts: the single agent tracking problem and the multi-agent scalability problem.

### 2.2. Single Agent Tracking Problem

To achieve the control objective, each vehicle must reach a given speed while maintaining a predefined desired spacing δ>0 from its predecessor. For simplicity in the analysis, the same δ is used for every pair of vehicles. The leader (i=1) travels in an independent fashion at a constant speed whenever possible. If the platoon is adequately designed, the vehicles will reach the speed of the leader in steady state, achieving the desired formation enforced by δ. In other words, under no disturbances we will have that ${\dot{x}}_{i}\left(t\right)={\dot{x}}_{1}\left(t\right)$ and $\ell_{i}\left(t\right)=\delta$ for all i>1 as t→∞. Notice that the position $x_{i}\left(t\right)$ in steady state should be a ramp signal. The single-agent problem is then to design a controller to achieve such tracking. This of course will depend on the available data at the controller side.

Linear control theory establishes that, if we want to track a ramp signal in a one d.o.f. control loop, two integrators must be placed in the open loop transfer function. Given the vehicle dynamics, the LTI model *H* has at least one integrator and, thus, the zero steady state error tracking can be achieved requiring local controllers to have integral action. Hence, simple local controllers as PID or PI may be sufficient to design a sensitivity function T=HK/(1+HK), where *K* is the controller, having an acceptable tracking performance. However, depending on the available information and the control scheme, more sophisticated control strategies can be adopted.

### 2.3. Multi-Agent Scalability Problem

This problem is related to how the whole platoon behaves when more vehicles are added. To illustrate the scalability problem, consider a naive control strategy in which each local controller only uses the positions $X_{i-1}$ and $X_{i}$ as

(2) $$\begin{array}{c} U_{i}=C_{p}\left(X_{i-1}-X_{i}\right) \end{array}$$

As the vehicles posses mass, integral action is needed for the platooning goal, and each local loop will contain two integrators. According to [39], such closed loop systems will satisfy

(3) $$\begin{array}{c} \int_{0}^{\infty }\ln\left|\frac{C_{p}\left(j\omega \right)H\left(j\omega \right)}{1+C_{p}\left(j\omega \right)H\left(j\omega \right)}\right|\frac{d\omega }{\omega^{2}}\geq 0, \end{array}$$

which implies that $T=C_{p}H/\left(1+C_{p}H\right)$, the complementary sensitivity function of the local closed loops, will satisfy ${\left||T|\right|}_{\infty }>1$, i.e., there exists ω≥0 such that |T(jω)|>1. For this simple interconnection topology of the agents, it has been determined that the effect of disturbances at the leader will affect the inter-vehicle spacing of the *k*-th follower through a transfer function possessing the term $T^{k}$ [9]. Consequently, disturbances affecting any vehicle will be amplified as they propagate along the platoon, which can become unsafe or inefficient if the string size *N* grows large. This behavior is commonly known as string instability [9].

To avoid string instability, two alternatives are (i) to use predecessor tracking with a constant time-headway spacing policy and (ii) to use a leader-predecessor tracking scheme with a constant spacing policy. In the following we present these two strategies; however, there are other approaches that can also be studied in the proposed experimental platform of this work.

#### 2.3.1. Predecessor Tracking with a Constant Time Headway Spacing Policy

An alternative to achieve a *string stable* platoon is to set [40]

(4) $$\begin{array}{c} U_{i}=C_{p}\left(X_{i-1}-X_{i}-hsX_{i}\right), \end{array}$$

where h>0 is a parameter and $C_{p}$ is a stabilizing controller for the model *H*. The parameter *h* is the *time headway* constant and this strategy can achieve string stability for all $h>h_{\inf}$ by changing the spacing policy δ of the scheme from a constant to a constant time headway. Assuming the vehicles starting at rest in the desired formation, under no disturbances we will have that ${\dot{x}}_{i}\left(t\right)={\dot{x}}_{1}\left(t\right)$ and $\ell_{i}\left(t\right)=\delta +h{\dot{x}}_{i}\left(t\right)$ for all i>1 as t→∞, where δ is not fixed in advanced but depends on the agent speed.

This control scheme maintains a simple communication topology but reduces some of the potential benefits that traveling with small pre-defined inter-vehicle spacings can yield [14].

#### 2.3.2. Leader-Predecessor Tracking with Constant Spacing Policy

Another alternative to achieve string stability is to use information of the leader. For instance, the output of the local controllers for i>2 can be chosen to be [10]

(5) $$\begin{array}{c} U_{i}=C_{p}\left(X_{i-1}-X_{i}\right)+C_{v}s\left(X_{1}-X_{i}\right), \end{array}$$

where $C_{p}$ and $C_{v}$ are controllers that stabilize *H* in closed loop. It can be noted that these control outputs are built by using the distance between an agent and its immediate predecessor and comparing the local velocities to the one of the leader.

This strategy enables the use of a constant inter-vehicle spacing policy given by a distance δ; however it requires the leader to broadcast its velocity to the rest of the platoon, which makes the communication topology more complex (and costly) as the number of agents increases.

### 2.4. Practical Aspects

Each agent should have several sensing capabilities, in order to apply the previous analytical results in a real setting. An experimental setup capable of replicating the behavior of a string stable control scheme requires local measurements, actuators, and communication and processing capabilities. This imposes practical constraints to study platooning in an experimental platform. Indeed, some of the above assumptions may not be satisfied in a practical scenario. However, some of the assumptions are not critical and others can be satisfied with a proper platform design.

For instance, considering agents with no volume as in the previous subsection does not invalidate the analysis or its application in a practical setting, as the relevant information needed for each agent corresponds to the distance from its frontal part to the rear of the predecessor, which can be measured with ranging sensors. The assumption that the agents travel in a one dimensional track can be satisfied with a suitable design of the workspace. Also, the coordination and automation of an arbitrary large number of agents cannot be implemented due to communication, processing and space constraints. However, a proper design of the experimental platform should allow us to work with sufficiently large platoons that exhibit the desired scalability behaviors of interest.

**Remark** **1.** *Here, we want to remark that for correctly exhibiting the scalability properties of a platooning configuration, a substantial number of agents may be needed. This, together with commonplace budget constraints (within the academic environment and even more so in developing countries) motivate the design of the proposed experimental setup, as acquiring the required number of agents from established vendors and brands is likely to be prohibitive. In the following sections we document the component selection for the agents, adding emphasis on budget reduction, ease of access and configuration, while ensuring that the requirements are met for these simplified platooning architectures of interest.*

## 3. Materials and Methods

To have an experimental setup to study platooning using the control algorithms described in the previous section, it is needed to design the agents and their workspace. The proposed platform is inspired by the dynamics of a train, but assuming that the agents (scale down train carriages) are not physically connected. In the following subsections we specify the technical capabilities, the component selection, functional modules and cost for the experimental platform.

### 3.1. Required Capabilities

To replicate a platooning scenario previously described, the vehicles should move in a one direction track with no overtaking. For implementing the specific control strategies mentioned in Section 2, every agent must be able to measure their own speed and sense the distance to its immediate predecessor. The leader must be able to broadcast its speed to every follower. The followers in turn, must be able to receive this information and make use of it. This must be done wirelessly, hence, communication capabilities that ensures proper transmissions and receptions on each agent is required. Also, each agent must have processing capabilities in order to autonomously control their own movement, based on the available information. An additional secondary requirement, is the capability for supervision and remote configuration of the agents. These must be implemented wirelessly by an external device. Finally, each agent must be low-cost, which makes increasing the number of agents affordable.

### 3.2. Platform Components

#### 3.2.1. Workspace of the Agents

**Figure 2:**
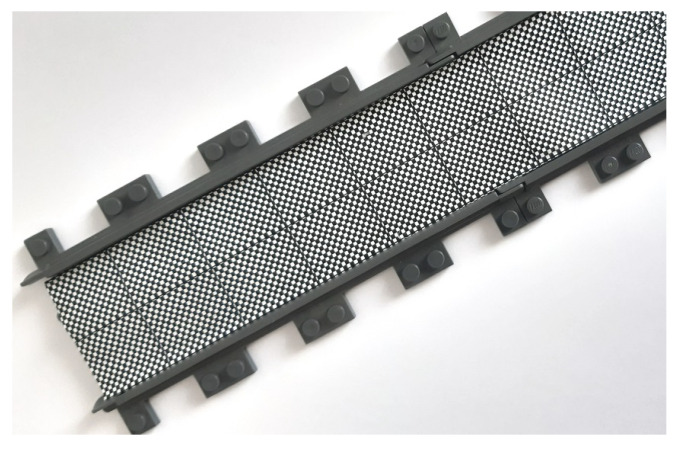
Tracks for the trains with checkerboard pattern for speed sensing.

Agents must be designed so that they move in one dimension, without overtaking. In our design, this is achieved by using small joinable LEGO^®^ rails to form a track on which agents move. The main track is a straight rail line; however, given the flexibility of the LEGO^®^ rails, the track can be modified adding curved rails sections. With this design, it is not necessary to include control algorithms to keep the agents properly placed and oriented in the track, and the platoon analysis can be focused on the main problems described in Section 2, i.e., longitudinal formation control and string stabilization. Each rail surface has a checkerboard pattern (see Figure 2), which is used for enabling the speed sensing.

#### 3.2.2. Processing Devices

**Figure 3:**
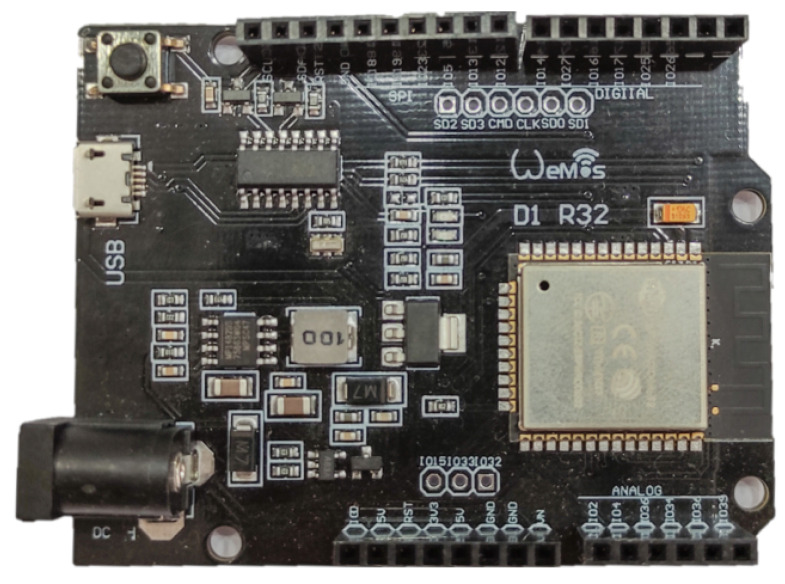
Arduino format ESP32 MCU board.

For each agent we chose an Arduino format board including the ESP32 System on a Chip (SoC) micro-controller [34,41], developed by *Espressif*, as the main computing unit, due to its capabilities (see Figure 3). The ESP32 has a dual core Xtensa LX6 CPU@240 MHz and a wireless communication module integrated in the chipset of the device, allowing the use of WiFi and Bluetooth Low Energy (BLE). It also possesses 34 general-purpose input/output (GPIO) pins for different functions as digital-only, analog-enabled, capacitive-touch-enable, and others. An important reason for this choice is that this is a very low-cost MCU, which is one of the main goals of this project.

For monitoring purposes, an extra processing device is also considered in the platform design. We use a Raspberry Pi 3 model B+ as a coordinator device which generates a WLAN and other capabilities of the platform. However, as the platform is designed and built with open hardware and software, any low range laptop or PC with a wireless adapter should be capable of working as the coordinator device.

#### 3.2.3. Actuation Devices

**Figure 4:**
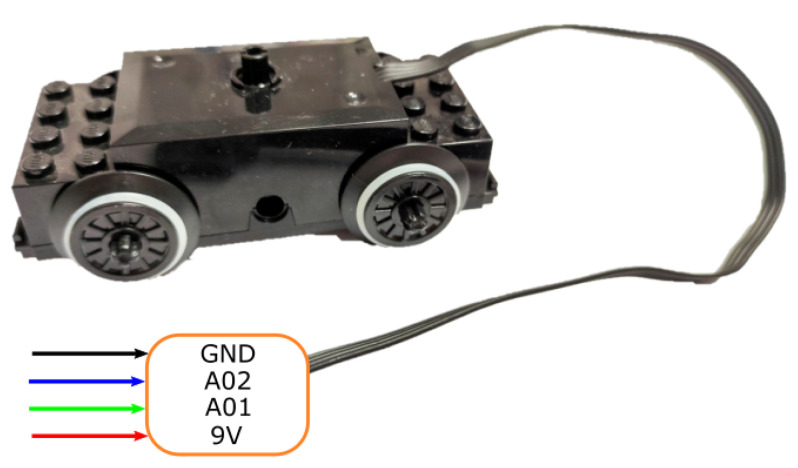
LEGO^®^, Motor wiring diagram.

**Figure 5:**
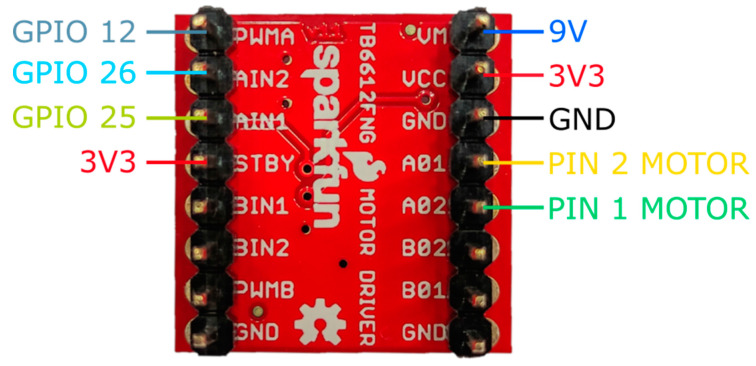
H-Bridge wiring diagram.

Each agent is equipped with a LEGO^®^, 9V DC motor (see Figure 4) controlled by an H-bridge driver. This motor, including wheels, is selected due to its compatibility with the physical design of the rails in the workspace. The used H-bridge corresponds to a TB6612FNG breakout board (see Figure 5), able to deliver an average current of 1.2 [A] and tolerating a peak of 3.2 [A] to two motors. In our design, only one channel of the integrated circuit is used, occupying 3 GPIOs of the micro-controller board: two pins to establish the direction of the rotational movement of the wheels and one pin for the PWM signal generated by the control algorithm within the MCU. The driver is tasked with processing the PWM signal generated by the micro-controller to manipulate the position and/or velocity of the agents through an external power source.

#### 3.2.4. Sensing Devices

Each agent has two sensors, the VL53L0X and ADNS3080 breakout boards, which are in charge of measuring the distance respect to its predecessor and their own velocity respectively.

**Figure 6:**
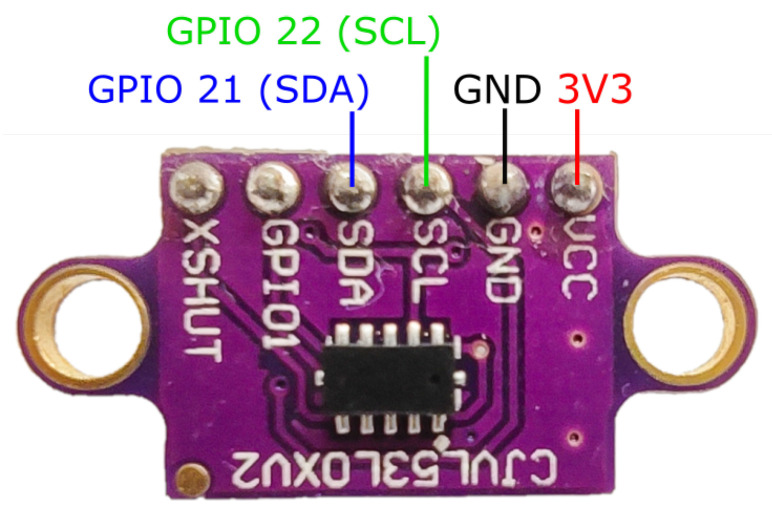
Distance sensor wiring diagram.

The VL53L0X [42] device is a *Time-of-Flight* (ToF) ranging sensor (see Figure 6) which is set in the front part of each agent, and is used to measure the distance to the predecessor agent. These modules measure distance based on the time for emitted photons to be reflected by the nearest object. The sensor uses the I${}^{2}$C communication protocol to interact with the MCU and can measure distances up to 200 cm in its fast measurement setting.

**Figure 7:**
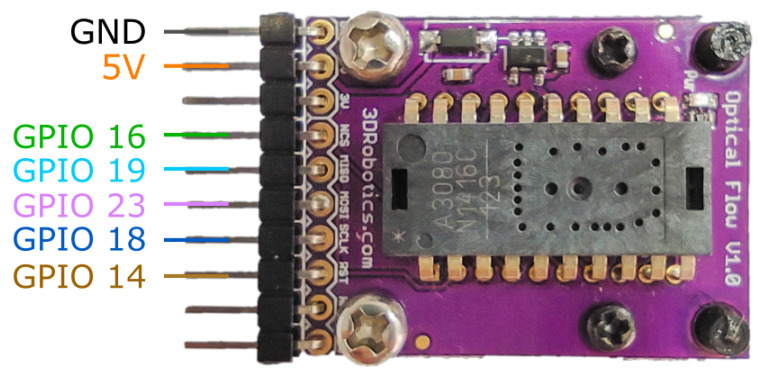
Velocity sensor ADNS3080 wiring diagram.

On the other hand, to measure the agent velocity we use an approach based on the ADNS3080 optical flow sensor module (see Figure 7), used in optical mice. The sensor is programmed/configured by writing into its registers through an SPI interface, and processes the captured images of the surface. Each optical sensor is able to capture images at a rate up to 6400 frames per second. We locate the sensor in the rear of each agent, capturing images of the pattern on the surface of the track. From there, by knowing the time between measurements and the bi-dimensional displacement provided by the sensor, we can estimate the instantaneous velocity of the agent after a calibration procedure has been performed. We add an LED light located next to the camera to improve the performance of the sensor.

It is worth mentioning that, in our setup, the ADNS3080 sensor can also provide measurements of the agent acceleration, if needed.

#### 3.2.5. Power Supply

Each agent is equipped with a 9 [V], 600 [mAh] rechargeable battery, to supply energy to the micro-controller board (which includes the communication module), the DC motor through the H-bridge circuit, and indirectly to the ToF sensor, the optical sensor and the LED light. With this battery, the agents are able to function autonomously long enough to carry out several experiments with wireless communication enabled.

#### 3.2.6. Chassis: Design and Assembly

**Figure 8:**
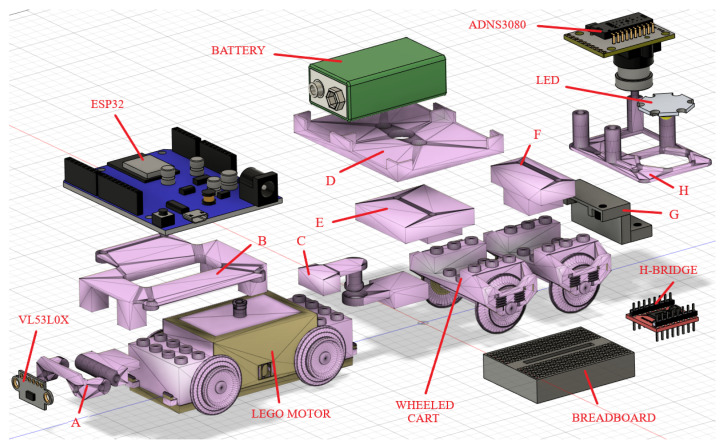
Disassembled PL-toon agent.

The chassis design for a single agent begins with the LEGO^®^, 9V DC motor. We designed 3D printed pieces that can be attached to the motor and are also capable of carrying and securing the different sensors and needed circuitry (see pieces A-H in Figure 8). The main component of the agents, which is attached over the motor, is the ESP32 micro-controller board, while the driver circuit/power stage (H-bridge), a LED, the 9 [V] DC rechargeable battery, and speed sensing camera are positioned over a secondary wheeled part, joined to the back of the LEGO^®^, motor with 3D printed pieces (see Figure 8). This joint can rotate with little friction, allowing the extended train to travel over curved tracks with no problems (C in Figure 8). This design choice also permits to lower the center of mass of the train with respect to its length, which provides stability to the agents. At the front of the agents, a small 3D printed connector enables attaching the ranging sensor and to set the angle with respect to the surface to obtain proper measurements of the immediate predecessor or obstacles on the way.

The assembly of each agent is straightforward as the diagram in Figure 8 shows. Firstly, most of the 3D printed pieces are assembled through screws over the motor and the wheeled back cart. These pieces are designed specifically for the components to be used. The micro-controller is then fastened over the piece that fits on the motor (B in Figure 8). The 9V battery and a mini breadboard that holds the H-bridge are fastened over a piece that fits on the back cart (D in Figure 8). The distance sensor is attached to the 3D part designed for this purpose in the front of the carriage and needs to be adjusted to aim correctly at the front of the agent (A in Figure 8). The ADNS3080 sensor is fastened with an LED light to a piece attached to the back of the secondary cart (H in Figure 8). Finally, all the wiring is made according to the diagrams in Figure 3, Figure 4, Figure 5, Figure 6 and Figure 7.

#### 3.2.7. Overall View of the Platform Design and Costs Breakdown

**Figure 9:**
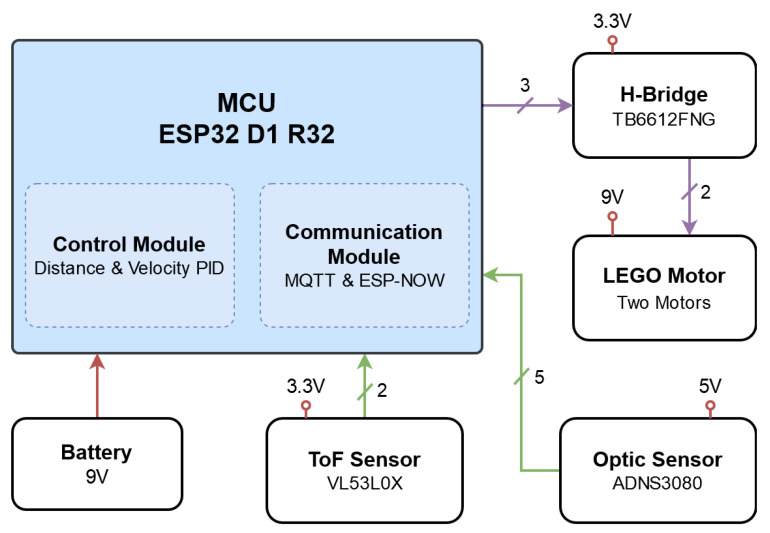
Block diagram of a single agent. Every agent is composed of the same modules and sensors.

**Figure 10:**
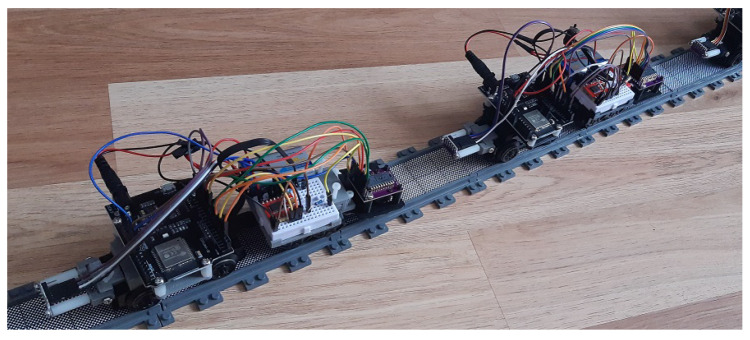
PL-Toon experimental platform.

In Figure 9, a block diagram depicts the interaction of the previously described electronic components. All these components are incorporated in the designed chassis as described in the previous section. Figure 10 present the platform design with real components. The software used with the platform is discussed in the next subsection.

Table 1 shows the approximate costs of the components chosen for each agent. The tracks, the coordinator unit (Raspberry Pi) and any other extras will add from $50 to $75 USD to the cost of the platform depending on the size of the workspace and the number of agents to be used.

### 3.3. Software

#### 3.3.1. Programming Languages and Environments

Within the software development area of the platform we can identify two kinds of programming: internal and external programming of the platoon. The first refers to all the coding needed for the correct functioning of the platform, while the second corresponds to programmed apps that are used to interact with the platform and/or individual agents, but have no effect in their fundamental behavior.

The internal programming considers mainly the MCU coding, which can be easily done using the Arduino IDE. The ESP32 libraries needed for this interaction are mature enough and such that most Arduino functions and peripheral interactions work with little effort. Register configurations of the sensors, and wireless communication initialization are all done in the setup area of the code. More importantly, a calibration stage is performed every time the agents are powered on.

For the agents, we use two communication protocols supported by the MCU: the Message Queuing Telemetry Transport (MQTT - ISO/IEC 20922) and ESP-NOW, a WiFi protocol developed by Espressif. In the case of MQTT, the open source Mosquitto client is used in either a Raspberry Pi, or in a computer connected to the same WLAN as the agents. Additionally, to capture and monitor the data from experiments, a laptop with a wireless adapter capable of monitor mode is required. A program developed in C++ (based on https://github.com/thomasfla/Linux-ESPNOW, accessed on 15 September 2020.) is used to capture the wireless ESP-NOW packets sent by the agents in a Linux machine. With the same program, the distance and velocity measurements from each agents are stored in a CSV file for further analysis or real time plotting with Python or any other method. Internal parameters of the agents such as PID gains, sampling times, etc. can be modified at the corresponding MQTT topic, from any MQTT enabled device (laptops, desktop PCs or handheld devices) given a suitable interface or app. We also design a simple dashboard allowing users to easily interact with the agents. It requires a Python interpreter in a computer and any device that can navigate with a browser will be able to observe a plot of the measured distance by the distance sensor of a single agent, modify the PID parameters or the desired reference distance to an obstacle, modify the frequency of the PWM signal etc., everything in real time. The measurements are also stored in a comma-separated values (csv) file for further manipulation and analysis. Fully functional codes for the agents and external devices, dashboard, 3D printing designs, and audiovisual material of the platform in action are provided in the repository of the platform https://github.com/pl-toon/pl-toon-codes (accessed on 10 March 2021).

#### 3.3.2. Functional Programming Modules

In this section we will present the modules that enable each agent of the platoon to function as an autonomous vehicle. We classify the programming of the platform within 3 functional modules: 1. Communication with an external device, 2. Inter-vehicle communication and 3. Sensing and Control. Their goals are self-explanatory, and we provide a few examples of the algorithms that can be found within them. 

**Communication with an external device:** Communication with a non-agent device is not critical for the fundamental tracking problem and functionality, but it is highly desirable for two main reasons: firstly, to allow the supervision of the internal state of the agents, as they transmit their sensor measurements and control actions to the external device for monitoring and processing; and secondly, to provide remote access for easily setting the controller coefficients, the time-headway constant or any other relevant internal agent parameters.

These tasks are implemented using the MQTT protocol. The publish/subscribe MQTT network protocol is widely used in IoT applications for being lightweight and easy to configure. This protocol requires a dedicated device, acting as a *broker*, for coordinating the requirements and messages of all the clients (agents). For the current setup, this job can be given to either a Laptop with a wireless adapter, a Raspberry Pi 3 B+ (two alternatives that we tested) or any similar device.

Algorithm 1 is an example of the routine to modify the controller parameters via MQTT. At the start of the main loop, the agents check for updates within the subscriptions. After the topic is matched, the corresponding internal variable of the agent is updated and the code resumes execution.
**Algorithm 1** Example of MQTT routine to change PID parameters.1:Receive message from topic2:Convert the number in message to a floating point value3:**if**$topic==K_{p}$**then** Set $K_{p}$ to value4:**end if**5:**if**$topic==K_{i}$**then** Set $K_{i}$ to value6:**end if**7:**if**$topic==K_{d}$**then** Set $K_{d}$ to value8:**end if**

**Inter-vehicle communication:** The platform requires the use of a communication protocol so the agents can interchange their measurements to do control afterwards. We use the protocol ESP-NOW [34], which is a wireless communication protocol developed by the manufacturer of the ESP32 board. Unlike MQTT, ESP-NOW does not require a router to establish a communication link. The protocol uses the 2.4 GHz channel band and the connection is similar to the one used by wireless mice.

The main reason for using this protocol instead of MQTT for the inter-vehicle communication is because ESP-NOW produces significantly smaller latency (less than 1 (ms)), so the transmitted data can arrive at the agents sooner, allowing the controller sample time to be reduced for a better performance and enabling real-time (for the agent dynamics) monitoring.

Algorithm 2 illustrates the ESP-NOW implementation when the leader broadcasts its speed for the followers to use in a cooperative adaptive cruise control implementation. When an ESP-NOW package is received by an agent, a check is performed to confirm if it was sent by the leader using its media access control (MAC) address. If this check is successful, the payload is stored and the leader velocity is extracted to update the corresponding internal variable of the agent, which is now available for updating the control signal.
**Algorithm 2** ESP-NOW routine for following agents.1:Receive the payload and the macAddress from the sender agent2:**if** macAddress≠macLeader **then return**3:**else**4:    Copy the payload bytes to a data packet structure5:    Set the global variable velocity_leader to the value received from the payload6:**end if**

**Sensing and Control:** In our setup, every agent is capable of measuring its own velocity and the distance to its predecessor. Furthermore, each agent has access to some of the measurements of other agents through the communication capabilities of the MCU. All this information is used to implement a functional module dedicated to control the movement of the agents. We consider that the code for processing the sensor data, the control signals calculation, and signal conditioning for the actuating devices, are all part of the control module.

There are basically two strategies to compute the control signals: centralized and decentralized [43]. In the first strategy the measurements of the whole platoon are used to calculate the control signals for each agent considering the global state of the platoon. This can be done in one processing unit, such as an external processing device. Therefore, once calculated in the external device, the control signals are sent through the communication modules to each agent. Clearly, this strategy undermines the autonomy of the agents. Conversely, in the decentralized strategy the control signals are calculated in the local MCU of the agents but using a subset of the platoon data, commonly data from local sensors and some received broadcasts from other agents. The communication network topology is expected to be simpler in a decentralized approach.

In our setup, the platoon controller is composed of a set of local controllers, implemented in the MCU of each agent that provides local control signals based on the available measurements. We assume a decentralized approach where each agent has access to some of the measurements of other agents, mainly the predecessor (through the ranging sensor) and the leader (through the ESP-NOW protocol).

To illustrate, we now present a routine to implement a PID-based control; however due to the versatility of the platform different control techniques can also be implemented. The particular implemented approach is composed by two PID controllers, and uses the agent velocity, the distance to its predecessor, and the leader velocity. The first PID regulates the difference between the measured and a desired distance, whereas the second PID is set up to maintain the respective agent velocity equal to the leaders velocity.

To implement the controllers we used the *Arduino PID Library*, which was chosen to its simplicity. To calculate the output value of a PID controller the library requires an error variable, a reference value and the PID gains. We use an additional variable α as a weight, allowing handling the relative importance between the two PID outputs. Algorithm 3 is done in every iteration of the main loop in the MCU:
**Algorithm 3** Control routine for following agents.1:Calculate the measurements of the distance sensor *d* and the velocity sensor *v*2:Calculate the errors $e_{d}$ and $e_{v}$ respect to the set-points3:**if** time since last call is bigger than SampleRate **then**4:     Compute $u_{d}$ with the distance PID and $e_{d}$5:     Compute $u_{v}$ with the velocity PID and $e_{v}$6:**end if**7:Calculate the motor input as $u=u_{d}+\alpha u_{v}$

Where $e_{d}$ and *u* are used later in the motor routine. The PID gains can be easily tuned within the Arduino IDE as well as remotely via MQTT. We should mention that this remote configuration can also be made through handheld devices with appropriate software.

## 4. Platform and Agents Set Up

Once an agent is assembled and programmed, for instance using the base codes provided in the repository of the platform, the set up of the platform can be done. In the following we summarize the functionality of the agents with the provided codes. At the startup, a self-commissioning is carried out by the agents in an automated fashion with the aid of the sensors, facilitating and speeding up the configuration of the agents for a new user.

### 4.1. Motor Dead-Zone Identification

The first step of is to detect the width of the motor dead-zone. For this task we use the ADNS3080 sensor. With a simple loop, the MCU slowly increases the duty cycle of the PWM signal applied to the 9V motor starting from rest. Once the optical sensor registers movement, the process is stopped and the corresponding value is stored for a proper implementation of the control algorithm, ensuring that the PID output is always over the dead-zone threshold whenever it requests the agent to move. As several factors can affect this value (such as battery levels, unexpected obstacles on the tracks, etc.) this process is performed each time the agents are switched on.

### 4.2. Sensor Calibration

To locally estimate the agent’s velocity, a calibration of the optical sensor is done after identifying the dead-zone. First, using the distance sensor as the reference for the calibration, the agent measures the distance to a standing object. Then the train moves forward and measures its distance to the same object again and does a comparison between the distance traveled measured by both the VL53L0X and ADNS3080. This is then used as a scale factor for future measurements of the optical sensor. The calibration process is done every time the MCU is powered on to guarantee consistent measurements between both sensors and also to make them independent of external factors such as the iteration of the experiment, the battery level and luminosity conditions of the environment.

### 4.3. Communication Initialization

Finally, the agents coordinate within both communication protocols. For the MQTT protocol, each agent subscribes to predefined *topics*, referring to relevant quantities (PID parameters, sampling rate, desired control scheme, etc.) or topics used for synchronization purposes. For the ESP-NOW protocol in broadcast mode (as for instance when the leader is the only agent sending date, while the followers listen) the setup is simple. A more involved preliminary step would be required for other communication topologies as the agents require the MAC addresses of their peers.

### 4.4. Remote User Interaction

Once the agent finishes the set up, it will stay on hold waiting for a start command on the MQTT broker. The reference between agents, the PID parameters, and other internal values, can be changed remotely in real time. After the agents start to use the PID control to regulate their distance to their nearest predecessor or obstacle, their measurements, control signals and other values are sent for supervision. The agents can be also stopped remotely through MQTT messages.

## 5. Comparison with Other Experimental Platforms

Similar platforms for teaching and research activities have been reported in the literature. For instance, the recent work [19] reports the development of an open source differential robot (MONA) possessing proximity sensors but lacking wireless communication capabilities in its most basic version. An expansion module can be used to implement inter-agent communication, taking the cost per agent over the $100 USD mark. In [21] (microMVP) and [44] (Colias), two low cost robotic platforms are reported. They are used for the study of trajectory following and swarm research, respectively. The microMVP platform has a per agent cost of $90 USD and even though the agents posses wireless capabilities through the use of Zigbee modules, vehicles only receive data from a central control computer using a webcam for sensing the positions of the agents on the plane. In such a scenario the agents do not send any data, i.e., there is no inter-agent communication without further modifications. Agents in the Colias platform move on the plane and cost around $35 USD. They posses distance sensors and IR modules enabling communications with neighbors up to a 2 [m] range and are designed for research of large-scale swarming in a lab setting. As the agents are small and use a high gearbox ratio, there is no need to use a dynamic model and the differential-driven kinematics suffice. Other works such as [24] and [22] develop experimental platforms with agents capable of moving in 2 dimensions for implementing decentralized MAS. These platforms are capable of performing trajectory tracking, obstacle avoidance and multi-agent formation control. In both cases, the platforms rely on a hovering camera and a remote PC for supervision and computing of the control signals. The agents used in [24] have a cost over $4500 USD. Table 2 contains a general comparison with the mentioned and other platforms.

The platform proposed in this work considers agents whose dynamic model is easy to identify and also modify. For platooning applications and their development it is relevant to consider this aspect, as properties such as string stability and collision avoidance may only be studied through a thorough mathematical description of the MAS. Moreover, being able to modify the dynamic model of the agents (e.g., by changing the weight of the agents) allows for flexibility of the platform when used in PID control or a system identification lab experience. By simplifying the platform to the 1D case when moving on tracks, we avoid the need for a webcam for supervision and of a remote PC for centralized control. Each agent is fully autonomous and capable of broadcasting its state to any neighbors or central controller, but can be accessed if needed for remote configuration of its parameters in real time. The spatial distribution of the tracks can be made arbitrary large and limited only by the range of the wireless communications and ranging sensors, rather than by the field of view of a camera.

## 6. Results

In this section we report experimental results in the context of teaching activities to perform with the platform. Research activities are discussed in the next section.

For teaching activities, we consider two types: classroom experiments and lab experiments. In the first case, experiments are not space demanding and can be done in a classroom, where each student or a pair of students, manipulates and studies the movement of one agent. In the second case, more complex experiments can be carried out for a platoon of agents, but this should be done in a lab with appropriate dimensions.

### 6.1. PID Control Classroom Assignments

Proportional-integrative-derivative (PID) control is one of the most simple but important control techniques. This control mechanism is commonly studied in undergraduate control courses and is widely used in the industry. As stated in previous sections, PID controllers can be implemented in each agent of the platoon to maintain a desired distance between agents. In a classroom environment, single agent experiments can be carried out to teach PID control with minimal set up using the proposed platform of this work.

Below we present a set of laboratory assignments related to the study of PID control and then an specific description of the related activities. This activities are designed for two-students lab groups with the same background, which includes a course in linear systems analysis, and a previous or simultaneous course on control systems in which the theoretical concepts of PID control is studied. The main goal of this activities is to illustrate how an empirical PID tuned controller performs.

With the proposed activities the student should:

Assignment 1: Understand the main characteristics and operation of a single agent of platform.
Assignment 2: Determine suitable controller parameters applying empirical PID tuning techniques.
Assignment 3: Compare the performance of the controller for different PID parameters.

#### 6.1.1. Assignment 1

Students should familiarize themselves with the experimental platform. They must perform a superficial inspection of its components, and then interact with the platform through the monitoring software and dashboard, performing certain basic experiments. For this, students must: (a) Study the dashboard and its functions to obtain the sensor measurements, send control signals to the actuators, store this data and plot it, and to vary the controller parameters. (b) Keep the agent at a fixed distance from an fixed obstacle and plot the measured distance. Repeat this at different distances from the object and verify that the measurements are consistent with the observed distance. (c) Send control signals to the actuators to move the vehicle forwards and backwards, maintaining a certain safety distance from an obstacle. Plot the measurement of the control signal and the measured distance, and check that they are consistent. (d) Obtain the current controller parameters using the software, change them remotely, and then request them again to verify that these parameters were modified successfully.

#### 6.1.2. Assignment 2

**Figure 11:**
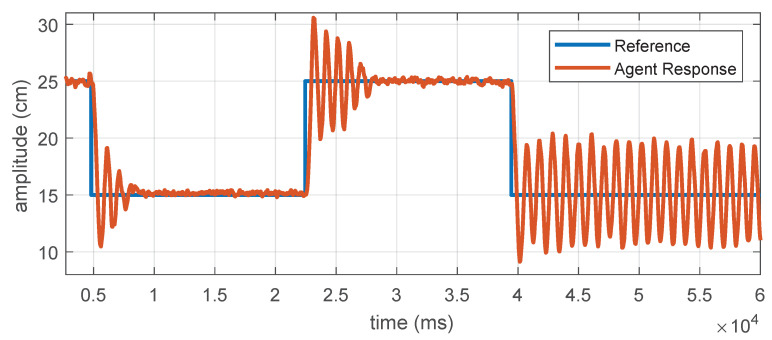
Plots for the Ziegler-Nichols second method experiments with a single PL-TOON agent.

Students are required to use the measured distance respect to a static object to run the well known *Ziegler-Nichols* second method for PID tuning. In Figure 11 we can observe a plot of such an experiment performed with an agent of the platform. The procedure is as follows: (a) Set $K_{i}=K_{d}=0$ and increase the parameter $K_{p}$ in the PID controller, and perform step changes on the desired distance to the static object (blue line) until the measured distance by the ranging sensor (orange line), exhibits oscillations in steady state. (b) Specify the critical value for the gain $K_{p}$, and estimate the frequency of oscillation from the collected data. (c) Use this information to compute a set of values for $K_{p}$, $K_{i}$ and $K_{d}$, according to the well-known Ziegler-Nichols gain estimator chart. (d) Test the tuned PID controller using step references and describing the transient and stationary system response.

#### 6.1.3. Assignment 3

**Figure 12:**
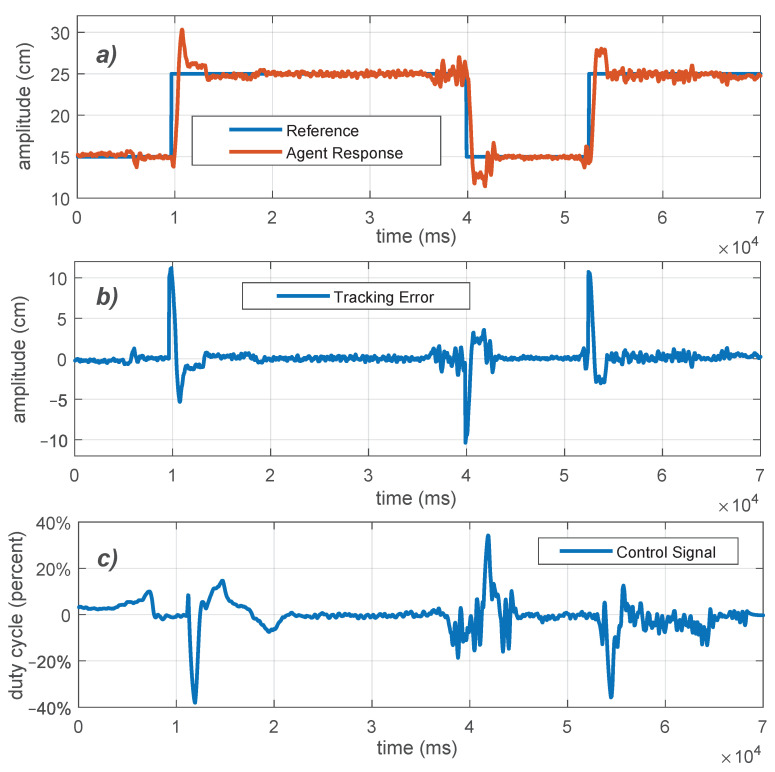
Experimental closed loop response of a single agent for step changes on the desired spacing to an obstacle using PI (the first 30 s) and PID control (the remaining time). (**a**) Distance reference and agent response. (**b**) Tracking error. (**c**) Control signal in terms of the duty cycle in percentage of the PWM signal (the sign defines the direction of movement).

The tracking performance of the PID controllers of a single agent can be evaluated running experiments and analyzing the system response. Given the monitoring capabilities within the platform, the rising time, overshoot, tracking errors an other information can be measured quantitatively, allowing for a proper performance evaluation. Additionally, having access to the position and velocity of the agents wirelessly, enables to observe quantitatively in *real time* how these measurements change in response to varying the PID parameters $K_{p}$, $K_{i}$, and $K_{d}$. Such values can be easily changed wirelessly and we can also evaluate the effect of turning off the derivative and/or integrative parts of the controllers. The plots of Figure 12 show the closed loop step response of a single agent with a PI (first step) and a PID controller (second and third steps) in one experiment, both tuned with the Ziegler-Nichols method. It can be noted that the derivative term provides a smaller overshoot, as it is predicted from classic linear control theory. However, the presence of noise in the output measurements becomes a drawback noticeable in the control action.

Specific activities are: (a) Set in the platform the parameters of a Ziegler-Nichols tuned PID controller and perform a step reference tracking. Store the control action, reference and measured distance. (b) Use the collected data to measure the rise time, overshoot, settling time and tracking error based on an Integral of the Square error (ISE) index. (c) Repeat (a) and (b) for different set of parameters, including the case of $k_{i}=0$, (PD controller), $k_{d}=0$ (PI controller). (d) Compare the perfomance of the controllers based on the measured rise time, settling time, overshoot and tracking error.

**Remark** **2.** *We want to highlight that the data to generate Figure 11 and Figure 12 were obtained by Electronic Engineering students in a remote laboratory at UTEM (Chile), using similar procedures as the ones we propose. Student feedback of the sessions was positive and most groups were able to successfully execute the tasks of Assignments 1 to 3 of this section.*

### 6.2. Platooning Lab Experiments

The experimental platform can be used to teach undergraduate and graduate students about string stability. This can be done based on well known results (See Section 2), where some control schemes are not always compatible with string stability.

We propose to use the two control approaches given in Section 2, namely, predecessor tracking with a constant time headway spacing policy and leader-predecessor tracking with a constant spacing policy. In both cases, a proper controller design yields a string stable platoon, but such design differs from one case to the other. In any case, the experimenters should be aware that the agents could collide for string unstable scenarios. Consequently, they should be prepared in case an agent leaves the tracks accidentally.

Below we specify the assignments for a string stability experience and describe the related activities. The experience is designed for 3 students teams with the same background, which includes a course in linear systems analysis, and a course on control systems and a previous or simultaneous course on advanced control systems where the notion of string stability is introduced.

With the proposed activities the students should:

Assignment 1: Understand the main characteristics and operation of the platform.
Assignment 2: Identify the concept of string instability via experimental results.
Assignment 3: Obtain a string stable behavior in a predecessor-tracking setup.
Assignment 4: Obtain a string stable behavior in a leader-tracking setup.

#### 6.2.1. Assignment 1

To carry out this assignment, students must perform the same activities as Assignment 1 in the previous subsection but adding wireless inter-vehicle communication. Thus, we add some steps: (e) Set a 4 agents platoon on the track, turn on the wireless inter-vehicle communication using the external device software and set it to send the leader velocity to the remaining vehicles in the platoon. (f) Move the leader, setting 30 % of the duty cycle for the motor. (g) Store and plot the leader speed data received in each vehicle and check whether it is concordant with the speed locally measured in the leader.

#### 6.2.2. Assignment 2

**Figure 13:**
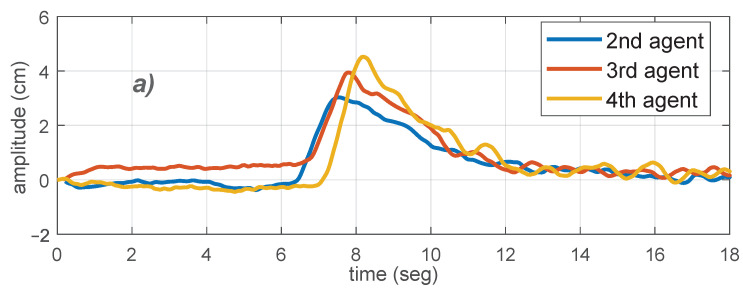

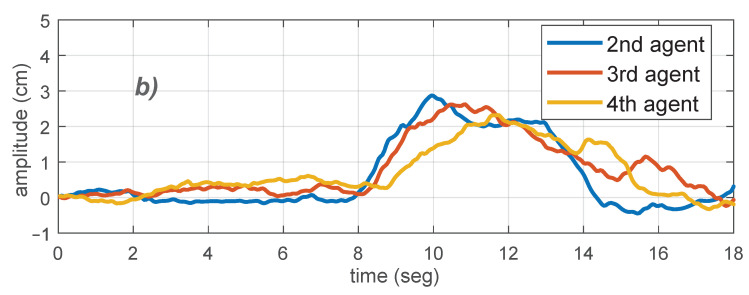
Experimental results for a four agents platoon, starting from rest to a constant speed. (**a**) Followers tracking errors for a string unstable scenario. (**b**) Followers tracking errors for a string stable scenario with time-headway constant.

Based on the results in Section 2, one way to observe string instability in a platoon is to consider a simple predecessor-following topology with no time-headway policy. The string unstable behavior can be observed at the beginning of the experiment starting from rest, and also for any abrupt change of the leader speed. An example of what is expected in this type of experiments is depicted in Figure 13, where experimental results were obtained using the proposed experimental platform with platoon of 4 agents. In the upper plot, the tracking error peaks increase along the chain of agents, which is a string unstable behavior.

Steps related to assignment 2 are: (a) Design a PID controller as in the previous subsection and set the corresponding parameters for each local controller. (b) Implement a predecessor-following topology as described in Section 2. This can be achieved using the local ranging sensor measurements. (c) With the platoon at rest, move the leader with a step signal for the speed and stop when the leader reaches the end of the track. During the experiment, collect data from each agent. (d) Plot the collected data and check a string unstable behavior.

#### 6.2.3. Assignment 3

In this assignment, a string stable controller design must be tested, verifying that agent errors are not amplified along the string. One way to do this is to use a constant time headway policy in the predecessor-following strategy. In Figure 13b, a strategy based on a constant time headway policy was implemented, leading to a string stable performance.

The tasks to achieve this assignment are: (a) Design a PID controller, for a given constant time headway *h* (see Section 2), such that the string stability condition ${\left||T|\right|}_{\infty }<1$ is satisfied for this topology. (b) Implement a predecessor-following topology with constant time headway as described in Section 2. Set the PID parameters and the constant *h* obtained in the previous task for each local controller. (c) With the platoon at rest, move the leader with a step signal for the speed and stop when the leader reaches the end of the track. During the experiment, collect data from each agent. (d) Plot the collected data and check if string stability is achieved. (e) Repeat points (a) to (d) for a higher value of the leader velocity. (f) Observe that increasing the leader velocity yields an increasing inter-vehicle distance due to the time headway policy.

#### 6.2.4. Assignment 4

For leader-predecessor tracking schemes, similar experiments can be carried out. In this case, however, the desired inter-vehicle distance is a constant value, so the leader speed can be increased or decreased and the steady state inter-vehicle distance must remain unchanged.

Steps to achieve this assignment are: (a) Design a PID controller, for a leader-predecessor tracking (see Section 2) such that the string stability condition ${\left||T|\right|}_{\infty }<1$ is satisfied for this topology. (b) Implement a leader-predecessor tracking setup as described in Section 2. This can be achieved using the local ranging sensor measurements and the leader velocity transmitted wirelessly to each agent. Set the PID parameters obtained in the previous task for each agent controller. (c) With the platoon at rest, move the leader with a step signal for the speed and stop when the leader reaches the end of the track. During the experiment, collect data from each agent. (d) Plot the collected data and check if a string stable behavior is achieved. (e) Repeat points (a) to (d) for a higher value of the leader velocity. (f) Observe that increasing the leader velocity does not affect the inter-vehicle distance, as expected.

## 7. Discussion

Platooning and string stability have been widely studied in the literature. However, a large majority of the contributions are theoretical- or simulation-based. The proposed platform was used successfully to implement cooperative platooning algorithms with open software and low cost components. Its affordability (in cost per agent) and flexibility allow for validation or easy prototyping of more involved platooning schemes, while enabling experimentation that complements simulation studies of practical implementations. The proposed platform is a well balanced alternative to perform teaching activities as those proposed in the previous section, but also for testing platooning theoretical results considering the effect of practical and technical aspects such as hardware and software limitations. Indeed, in recent years, practical issues in platooning have become relevant research topics. We now discuss some research applications of the platform.

### 7.1. Communication Network Aspects

Research experiments can be performed putting the communication network topology, and the information shared by the agents as main subjects. The stability and performance should depend on the kind of information shared between agents, such as speed, position, acceleration, control signals, etc. The network topology establishes which agents are transmitting and receiving certain data. There are many different possibilities to test on the platform by modifying the communication setup.

Communication impairments are also a relevant research topic. One can experimentally observe how communication delays, random data-loss, noisy channels and other communication issues affect the platoon performance. Delays and data dropouts can be emulated via coding or observed under certain conditions. A related experiment could be determining how many agents can be added to the platoon until a communication issue arises due to limited channel capacity. Another possibility is to measure how far the leader can broadcast its data to the rest of the platoon and test whether the speed of the agents plays a role.

### 7.2. Security in Cyberphysical Systems

Security issues are also an important research topic. Since the platoon may rely on a communication network, a critical aspect in a real application is ensuring transmitted data is not corrupted, changed or accessed by external attackers. There are several types of cyber-attacks against which agents must be protected. The platform allows for testing security algorithms and/or protocols that can be used for this needed protection.

### 7.3. Advanced Control Algorithms

Finally, it is important to highlight that not only PID control can be tested within the micro-controller unit. Indeed, more sophisticated control and estimation algorithms can also be implemented, such as Kalman filtering, linear quadratic regulator (LQR), model predictive control (MPC), etc. Clearly, the computational demand of the MCU must be considered given the unavoidable processing speed limitations. For instance, designing predictive control with long prediction horizon would become infeasible to implement. Nevertheless, the platform is designed to be flexible not only in the communication topology, but also in the control algorithms to be used for both teaching and research.

### 7.4. Platform Limitations

As the platform is built with low cost components, there exist some limitations on what the agents are capable of achieving. The ToF module VL53L0X can only measure up to 2 (m) with a precision nearing 5 (mm). Moreover, there are some strict timing constraints that limit the fastest sampling time to 20 (ms). Speed measurements with the ADNS3080 can achieve resoultions of 1 (cm/s) and detect speeds up to 100 (cm/s) with the timing constraints imposed by the ranging sensor. Both of these measurements have noticeable amounts of noise that can are managed with simple filtering routines (included in our routines at the repository). Abrupt changes of direction of the agents may lead to wheel slippage.

## 8. Conclusions and Further Platform Development

We presented an experimental multi-agent system platform for teaching of control labs and platooning. The design of the platform is open, low-cost, flexible and capable enough for the verification of cooperative platooning schemes.

The individual agents of the platform are also well suited for teaching of PID control labs (and embedded systems). Moreover, the agents can be accessed and supervised remotely, enabling remote experiences for very low costs, complexity and assembly time.

The platform was used as a teaching tool for remote laboratory modules, mitigating the effects of the 2020 pandemic at the Faculty of Engineering of Universidad Tecnológica Metropolitana, in Santiago de Chile.

Future works with the platform will include the development of an easy to use Graphical User Interface for remote labs and verification of other platooning control schemes. Novel research directions aided by the platform will consider the proposal of new control schemes with different spacing policies and interconnection topologies, and the study of the effects of lossy communications on the scalability properties of multi-agent systems.

We conclude by mentioning that it is reasonable to assume that more powerful MCU and better sensors in similar formats will be available in the future. Given the open design of the agents, updating the MCU or any other part, maintaining the basic functionality, should be possible, allowing extending the lifespan and improving the capabilities of the agents and the platform.

## Figures and Tables

**Table 1:** Component costs for an agent.

Component	Cost (USD)
ESP32	$4
LEGO^®^, Motor	$5
VL53L0X	$1.5
ADNS3080	$8
Mini Breadboard	$0.25
Battery	$4.5
Extras	$5
**Total**	$28.25

**Table 2:** Comparison of other non-commercial experimental platforms.

Robot	Release Date	Cost	Research Education	Inter-Agent Communication	Movement Coordination
Aeris [45]	2017	Unknown	Both	Unknown	Unknown
Andruino-A1 [46]	2016	39 USD	Education	Yes	Decentralized
Colias [44]	2014	35 USD	Research	Yes	Decentralized
MarXbot [20]	2009	Unknown	Research	No	Decentralized
μCar [23]	2020	480 USD	Both	Yes	Decentralized
microMVP [21]	2017	90 USD	Both	No	Centralized
MONA [19]	2018	100 USD	Both	No (add-on)	Decentralized
Rice r-one [47]	2013	250 USD	Both	Yes	Centralized
Thymio II [48]	2010	135 USD	Education	No	Decentralized
PL-Toon	2021	30 USD	Both	Yes	Both

## Data Availability

The data presented in this study are available in https://github.com/pl-toon/pl-toon-codes (accessed on 10 March 2021).
